# Chemical composition of 8 *eucalyptus* species' essential oils and the evaluation of their antibacterial, antifungal and antiviral activities

**DOI:** 10.1186/1472-6882-12-81

**Published:** 2012-06-28

**Authors:** Ameur Elaissi, Zyed Rouis, Nabil Abid Ben Salem, Samia Mabrouk, Youssef ben Salem, Karima Bel Haj Salah, Mahjoub Aouni, Farhat Farhat, Rachid Chemli, Fethia Harzallah-Skhiri, Mohamed Larbi Khouja

**Affiliations:** 1 Laboratory of Pharmacognosy, Faculty of Pharmacy, University of Monastir, Avenue Avicenne, Monastir, 5019, Tunisia; 2 Laboratory of Transmissible Diseases and Biologically Active Substances, Faculty of Pharmacy, University of Monastir, rue Avicenne, Monastir, 5000, Tunisia; 3 Laboratoire of Microbilogy, Imuunology, EPS Farhat Hachad, Sousse, Tunisia; 4 Laboratory of Analytical Chemistry, Faculty of Pharmacy, University of Monastir, Avenue Avicenne, Monastir, 5019, Tunisia; 5 Laboratory of Genetic, Biodiversity and Bio-resources Valorisation, Higher Institute of Biotechnology of Monastir, University of Monastir, Avenue Tahar Haddad, Monastir, 5000, Tunisia; 6 National Institute for Research on Rural Engineering, Water and Forestry, Institution of Agricultural Research and Higher Education, Ariana, BP, N.2, 2080, Tunisia

**Keywords:** *Eucalyptus* sp., Essential oil, Principal Components Analysis, Hierarchical Cluster Analysis, Antibacterial activity, Antifungal activity, Antiviral activity

## Abstract

**Background:**

In 1957, Tunisia introduced 117 species of *Eucalyptus*; they have been used as fire wood, for the production of mine wood and to fight erosion. Actually, *Eucalyptus* essential oil is traditionally used to treat respiratory tract disorders such as pharyngitis, bronchitis, and sinusitis. A few investigations were reported on the biological activities of *Eucalyptus* oils worldwide. In Tunisia, our previous works conducted in 2010 and 2011 had been the first reports to study the antibacterial activities against reference strains. At that time it was not possible to evaluate their antimicrobial activities against clinical bacterial strains and other pathogens such as virus and fungi.

**Methods:**

The essential oils of eight *Eucalyptus* species harvested from the Jbel Abderrahman, Korbous (North East Tunisia) and Souinet arboreta (North of Tunisia) were evaluated for their antimicrobial activities by disc diffusion and microbroth dilution methods against seven bacterial isolates: *Haemophilus influenzae*, *Klebsiella pneumoniae*, *Pseudomonas aeruginosa*, *Staphylococcus aureus*, *Streptococcus agalactiae*, *Streptococcus pneumoniae* and *Streptococcus pyogenes*. In addition, the bactericidal, fungicidal and the antiviral activities of the tested oils were carried out.

**Results:**

Twenty five components were identified by GC/FID and GC/MS. These components were used to correlate with the biological activities of the tested oils. The chemical principal component analysis identified three groups, each of them constituted a chemotype. According to the values of zone diameter and percentage of the inhibition (zdi, % I, respectively), four groups and subgroups of bacterial strains and three groups of fungal strains were characterized by their sensitivity levels to *Eucalyptus* oils. The cytotoxic effect and the antiviral activity varied significantly within *Eucalyptus* species oils.

**Conclusions:**

*E. odorata* showed the strongest activity against *S. aureus*, *H. influenzae*, *S. agalactiae*, *S. pyogenes*, *S. pneumoniae* and against all the tested fungal strains. In addition, *E. odorata* oil showed the most cytotoxic effect. However, the best antiviral activity appeared with *E. bicostata*. Virus pretreatment with *E. bicostata* essential oil showed better antiviral activity (IC_50_ = 0.7 mg/ml, SI = 22.8) than cell-pretreatment (IC_50_ = 4.8 mg/ml, SI = 3.33). The essential oil of *E. astringens* showed antiviral activity only when incubated with virus prior to cell infection. This activity was dose-dependent and the antiviral activity diminished with the decreasing essential oil concentration.

## Background

The *Eucalyptus*, a native genus from Australia, belongs to *Myrtaceae* family and comprises about 900 species [1]. More than 300 species of this genus contain volatile oils in their leaves. Fewer than 20, within these species, known for their high content of 1,8-cineole (more than 70%), have been commercially used for the production of essential oils in pharmaceutical and cosmetic industries [2]. Over the past few years, the interest in natural medicine has been increasing in industrialized societies particularly against microbial agents because of the ever growing problem of antibiotic resistance [3]. In Tunisian folk medicine, inhalation of *Eucalyptus* sp. essential oil has traditionally been used to treat respiratory tract disorders such as pharyngitis, bronchitis, and sinusitis [4]. Consequently, the scientific interest in this field has been expanding. Some researchers have demonstrated some efficacy of *Eucalyptus globulus* essential oil against *Haemophilus influenzae* and *Stenotrophomonas maltophilla*[3,5]. *Streptococcus pyogenes, Streptococcus pneumoniae, Streptococcus agalctia, Staphylococcus aureus, Pseudomonas aeruginosa, Klebsiella pneumonia* and *Hemophilis influenzae* are the most important causes of the respiratory tract infections and the most resistant to antibiotics. Many studies reported the antifungal propriety of plant extracts and essential oils against dermatophytes, filamentous and *Candida albicans*[6,7]. The essential oils extracted from *Eucalyptus* species, mainly from *E. urophylla* S.T. Blake, *E. grandis*, *E. camaldulensis*, *E. citriodora* and *E. globulus* were found to be active on phyto-pathogenic fungi [8-10].

Few studies have reported the antiviral activity of *Eucalyptus* essential oils against Adenovirus, mumps and herpes simplex viruses [11,12]. One of the major challenges is the practical use of these essential oils *in vivo*. This is difficult and needs to be overcome by trial assays. The importance of the antiviral activity of natural compounds is their perspective uses against these pathogens. Enteroviruses are more prevalent in the environment than the viruses discussed above and it is worth studying them for any antiviral activity. To the best of our knowledge, there is no published report on the *Eucalyptus* essential oils’ activities against human fungal and enteroviral infections. As reported previously [13,14], we have studied the antibacterial activity of 35 *Eucalyptus* essential oils against four reference gastrointestinal strains (*Esherichia coli,* ATCC 25922; *Pseudomonas aeruginosa,* ATCC 227853; *Enterococcus faecalis,* ATCC 292112; *Staphylococcus aureus,* ATCC 25932) using the disc diffusion method. On the basis of the best diameter inhibition against these pathogens, eight *Eucalyptus* essential oils were selected and used to evaluate their activity against bacterial strains isolated from patients suffering respiratory infections. In addition, these oils were tested for their antifungal and anti-enteroviral activities.

## Methods

### Plant materials

Samples of clean mature leaves of eight species of the genus *Eucalyptus* L’HÉR., five of which were collected in June 2006 from Souiniat arboreta located in the North west of Tunisia (*E. bicostata, E. cinerea*, *E. maidenii*, *E. odorata* and *E. sideroxylon*); two species were collected in April 2006 from Korbous arboreta (*E. astringens and E. lahmannii)*; one species was collected from Jbel abderrahaman arboreta (*E. leucoxylon*). The last two arboreta were located in Nabeul region (North East of Tunisia with sub-humid bioclimatic stage). Botanical voucher specimens have been deposited in the Pharmacognosy Laboratory Herbarium in the Faculty of Pharmacy (Monastir, Tunisia), under the following references: 0119, 0120, 0126, 0127, 0129, 0132, 0138 and 0154.

### Extraction of essential oils

The extraction of essential oils was carried out by hydro-distillation during four hours, using a standard apparatus recommended in the European Pharmacopoeia. We made three attempts for each sample of 100 g of boorishly crushed dried leaves for each species. The oil collected from each plant was then dehydrated with Na_2_SO_4_ (Sigma-Aldrich, NY, USA) and stored at 4°C until use.

### Chemical analysis

Quantitative and qualitative data for all the essential oils were performed in triplicate by Gaz Chromatography (GC) and Gaz Chromatography coupled with the Mass Spectroscopy (GC/MS), respectively.

### Gas chromatography analysis

GC was carried out using Hewlett-Packard (HP) 6890 chromatography apparatus equipped with Flame Ionization Detector (FID) and Carbowax column (30 m x 0.32 mm i.d., film thickness 0.25 μm) under the following analytical conditions: injector and detector temperatures were maintained at 250°C and 280°C, respectively; oven temperature programmed to rise from 35°C to 250°C at 5°C/min, isothermal temperature 35°C for 1 min and 250°C for 3 min; the carrier gas was nitrogen with a flow rate of 1.2 mL/min., the injected volume was 1 μL sample of 10% solution of oil in purified hexane. Relative concentrations were calculated using the software HP Chemstation, which allows the assimilation of the percentages of the peak areas to the percentages of the various constituents. Retention indices were obtained by running a series of aliphatic hydrocarbons (C9 - C28) by increasing the number of carbon atoms in the Carbowax GC column).

### Gas chromatography-mass-spectrometry analysis

The chemical analysis of the essential oils was carried out using a HP 5890 series II gas chromatography apparatus equipped with a polar column Carbowax (30 m x 0.32 mm i.d., film thickness 0.25 μm) and 5972 mass selective detectors. Helium was used as the carrier gas. The mass spectrometer operating conditions were: ionisation voltage, 70 eV, ion source 230°C. The GC/MS parameters were identical to those for the GC analysis.

### Compound identification

The identification of the compounds was based on the comparison of their retention index (determined relatively to the retention time of aliphatic hydrocarbons (C9 - C28) and of the mass spectra with those of authentic compounds by means of NBS75K.L. and Wiley 275 databases and with the literature data) [15].

## Antibacterial testing

### Bacterial strains

The bacterial strains used in the present study were seven clinical bacterial isolates: *Haemophilus influenzae* (11 strains), *Klebsiella pneumoniae* (13 strains), *Pseudomonas aeruginosa* (10 strains), *Staphylococcus aureus* (17 strains), *Streptococcus agalactiae* (9 strains), *Streptococcus pneumoniae* (19 strains) and *Streptococcus pyogenes* (2 strains). These clinical strains were obtained from Microbiology and Immunology Laboratory (EPS Farhat Hachad, Sousse, Tunisia).

### Kirby Bauer paper method

The antibacterial activity of the different essential oils was evaluated by the paper-disc agar diffusion method with a bacterial inoculum of 0.5 Mcfarland; Mueller-Hinton (MH) with 5% sheep blood or with MH only for *Pseudomonas aeruginosa, and Staphylococcus aureus.* Absorbent discs (Whatman disc n°3, 6 mm diameter) were impregnated with 10 μL of each oil, and then placed on the surface of inoculated plates (90 mm). Positive control discs of antibiotics commonly used for the treatment of respiratory tract diseases were tested. After 24 h of incubation at 37°C, the inhibition zones were measured and expressed in mm. All experiments were done in triplicate.

### Determination of MIC and MBC

The minimal inhibition concentration (MIC) was studied only with oils which were proved to effective using the disc diffusion method (inhibition zones ≥ 17 mm). MIC was determined using micro-well dilution method according to the protocol of Şahin et al. (2004) [16]. The 96-well plates were prepared by dispensing into each well 95 μL of nutrient broth and 5 μL of the inoculum. One-hundred microliters from each extract were initially prepared at a concentration of 0.166 (v/v) and added into the first well, followed by two-fold dilution until the 9^th^ well. The wells of column 10 were filled with 195 μL of MH broth and reserved for the bacterial growth control, whereas the 11^th^ column wells were reserved for the control of the broth sterility. The wells of the last column were used as a negative control, and contained 195 μL of nutrient broth and 5 μL of the inoculum. The plates were screened visually after incubation at 37°C for 24 h for broth turbidity. The minimum bactericidal concentration (MBC) is the lowest concentration of the essential oil that can kill 99.9% of the bacterial population after incubation for 18–24 h at 37°C [17,18]. It was calculated by inoculating the content of the well indicating the MIC and the wells that precede it in an agar plate.

## Antifungal testing

### *Fungal* strains

Seven *Eucalyptus* essential oils were tested against five fungal strains that comprise one opportunist pathogenic yeast (*Candida albicans*), one filamentous (*Scopulariopsis brevicaulis*) and three dermatophytes (*Trichophyton rubrum*, *Trichophyton soudanense*, *Microsporum canis*). The micro-organisms were obtained from the Laboratory of the Transmissible Diseases and Biological Active Substances LR99ES27 (Faculty of Pharmacy of Monastir, Tunisia).

#### Agar incorporation method

Antifungal activity was carried out by the agar incorporation method (dilution in a solid medium) including a negative control, as described previously by Bel Haj Salah et al. (2006) [19]. The test was performed in sterile Petri dishes (33 mm) containing Sabouraud Glucose Agar (SGA). Samples were mixed with ethanol 99% (v/v) to obtain a final concentration of 1000 μL/mL. This solvent was also used as a negative control. After cooling and solidification, the medium was inoculated with a small amount (5 mm) of a 7 day-old mycelium culture (for dermatophytes), a three days culture suspension adjusted to 10^5^ conidies/mL (*Scopulariopsis brevicaulis*) or a 3 day culture suspended in sterile distilled water and adjusted to 10^5^ spores/ml (for yeasts). The pevaryl was used as anti-fungal reference. The Petri dishes were then incubated for additional seven days at 24°C for dermatophytes and for the filamentous, 24 h at 37°C for Candida. Three replications were carried out for each concentration and for each micro-organism. The antifungal activity of the essential oils was evaluated by calculating the percentage of inhibition (% I) from the diameters of colonies in the control plate (dC) and the colonies in the treated plate (dE); % I = (dC - dE)/dC, according to the method of Singh et al. (1993) [20].

### Cytotoxicity assay

The evaluation of the cytotoxic effect of samples is based on the reduction of MTT (3-[4,5-dimethylthiazol-2-yl]-2,5-diphenyl tetrazolium bromide), by the mitochondrial dehydrogenase of viable cells, to give a blue formazan product which can be measured spectrophotometrically at 540 nm. The MTT colorimetric assay was performed in 96-well plates. Cells were seeded in 96-well plates at a concentration of 10^5^ cells per well and incubated for 24 h at 37°C in a 5% CO_2_ humidified atmosphere. After treatment with various concentrations of the test compound, the cells were incubated for an additional 48 h at 37°C. The cells were examined daily under a phase-contrast microscope to determine the minimum concentration of compounds that induced alterations in cell morphology. After that, the medium was removed and cells in each well were incubated with 100 μL of MTT solution (5 mg/mL) for 3–4 h at 37°C. Fifty microliters of dimethyl sulfoxide (DMSO) were then added to dissolve insoluble formazan crystal and the plates were incubated at 37°C for 30 min. Optical density (OD) was measured at 540 nm using a Perkin-Elmer ELISA reader (HTS 7000 plus). Data were obtained from duplicate wells. Cell viability was expressed with respect to the absorbance of the control wells (untreated cells), which were considered as 100% of absorbance. The percentage of cytotoxicity is calculated as [(A - B)/A] x 100, where A and B are the OD_540_ of untreated and of treated cells, respectively. The percentage of viability was carried out using the formula: 100 - % cytotoxicity. The 50% cytotoxic concentration (CC_50_) was defined as the compound’s concentration (μg/mL) required for the reduction of cell viability by 50%, which were calculated by regression analysis [y = f(x); where y = % viability and x = concentration of extract, μg/mL]. The used definition of the cytotoxicity, as supported by other reports [21] was; CC_50_ < 1.0 μg/mL – high cytotoxicity; CC_50_ = 1.0-10.0 μg/mL – moderate; CC_50_ = 10.0 - 20.0 μg/mL – mild cytotoxicity; and CC_50_ > 20 μg/mL – non cytotoxic.

### Antiviral activity

***Cell culture and virus.*** The Vero cell line was maintained in RPMI 1640 (Gibco, Tunisia) supplemented to fetal bovine serum (10%, v/v), L-Glutamin (2 mM), penicillin (100 U/mL), and streptomycin (100 μg/mL). Cells were incubated at 37°C in a 5% CO_2_ humidified atmosphere. Coxsakievirus B3 Nancy strain (kindly provided by Pr. Bruno Pozzetto, Laboratory of Bacteriology-Virology, Saint-Etienne, France) was propagated in Vero cells.

Confluent Vero cell cultures were treated with three non cytotoxic concentrations of the essential oil during and after virus infection in two sets of experiments as follows: (1) 5 x 10^4^ TCID_50_ of the virus was exposed to essential oil for one hr at 37°C. Then 100 μL of the mixture were added to the cells cultured fluently in 96-well flat-bottom microtiter plate (100 μL); (2) Cells were treated with essential oil (100 μL) for one hr at 37°C. After one hr of incubation at 37°C, 5 x 10^4^ TCID_50_ of the virus (100 μL) were added.

All plates were incubated at CO_2_-incubator for 48 hrs. The viability of the infected and non-infected cells was evaluated according to the absorbance values of formazan using the MTT inclusion assay, as described in cytotoxicity assay. The percentage of protection was calculated as follows:

Percent protection = [(ODT) - (ODC) V]/[(ODC) M - (ODC) V] × 100

Where (ODT), (ODC) V and (ODC) M indicate absorbance of the test sample, the virus-infected control (no compound) and mock-infected control (no virus and no compound), respectively. The 50% inhibition concentration (IC_50_) was calculated by regression curve analysis, which is defined as the concentration of the essential oil that inhibits the viral infection by 50% [22].

### Statistical analysis

The data were analyzed using analysis of variance (ANOVA). The significance of the differences between means was determined at *p* < 0.05 using Duncan's multiple range tests. Results were expressed as means ± Standard Deviation (SD). To evaluate whether the identified components may be useful in reflecting chemotaxonomic and biological activities relationships, 25 compounds detected in the oil samples at an average concentration greater than 0.9% of the total oil were selected and used for this purpose. Both of these components and all the values of the essential oils zone diameters of bacteria growth inhibition were subjected to a principal component analysis (PCA) and to hierarchical cluster analysis (HCA) using SPSS 12.0 software (SPSS Inc. Chicago, IL, USA). To evaluate the antiviral activity *in vitro*, the selectivity index (SI = CC_50_/IC_50_) was determined. The selectivity index describes the ratio between the cytotoxic and the antiviral activity of a tested compound.

## Results and discussion

### Chemical composition

The chromatographic analysis (GC retention index (RI) and GC/MS) of the essential oils allowed the identification of 144 components representing 87.40 to 99.37% of the total oil content [23-25]. Twenty five major compounds at an average concentration greater than 0.9 ± 0,2% have been retained for the statistical analysis (Table 1). The main components were 1,8-cineole (4.5 ± 1,61 -70.4 ± 2.5%) followed by cryptone (0.0 - 20.9 ± 1.3%), α-pinene (1.0 ± 0.7 - 17.6 ± 7.5%), *p*-cymene (0.8 ± 0.1 - 16.7 ± 5.2%), α-terpineol (0,6 ± 0,3 - 10,3 ± 1,1%), trans-pinaocarveol (0.8 ± 0.2 - 7.0 ± 2.5%), phellandral (0.0 - 6.6 ± 0.4%), cuminal (0.0 - 6.6 ± 0,6%), globulol (0.6 ± 0.2 - 6.2 ± 0.9%), limonene (0.4 ± 0.2 - 4.4 ± 0.3%), aromadendrene (0.1 ± 0.0 - 3.6 ± 1.2%), sapthulenol (0.1 ± 0.1 - 3.2 ± 0.9%) and terpinene-4-ol (0.3 ± 0.1 - 3.0 ± 0.8%). 

**Table 1:** **Contents (%) of the 25 major compounds of the essential oils, extracted from the leaves of 8* Eucalyptus* species, selected for the Principal Components and the Hierarchical Clusters Analyses**

**№**	**Compounds**	***Abbreviations***	***Content***^***a***^***(%)***							
			***E. maid***^***b***^	***E. cin***	***E. sid***	***E. odo***	***E. bic***	***E. leh***	***E. astr***	***E. leuc***
1.	α-Pinene	α-pin	7.3 ± 0.7	4.5 ± 0.7	6.9 ± 1.1	1.0 ± 0.7	3.7 ± 1.2	17.6 ± 7.5	22.0 ± 6.0	7.8 ± 2.3
2.	Limonene	lim	3.1 ± 0.2	3.7 ± 0.5	4.1 ± 0.1	0.4 ± 0.2	0.9 ± 0.5	4.4 ± 0.3	1.4 ± 0.3	2.0 ± 0.5
3.	1.8-Cineole	1.8-cin	57.8 ± 1.9	70.4 ± 2.5	69.2 ± 0.6	4.5 ± 1.6	68.0 ± 5.3	56.6 ± 4.3	42.0 ± 5.9	59.2 ± 10.1
4.	γ-Terpinene	γ-ter	1.7 ± 0.9	0.1 ± 0.0	0.1 ± 0.0	0.2 ± 0.2	0.1 ± 0.1	0.9 ± 0.2	0.1 ± 0.1	0.2 ± 0.4
5.	*p*-Cymene	p-cym	7.4 ± 2.9	1.2 ± 0.1	0.8 ± 0.1	16.7 ± 5.2	1.4 ± 0.5	2.0 ± 0.2	0.9 ± 0.2	2.9 ± 3.4
6.	Pinocarvone	pin	0.5 ± 0.2	0.3 ± 0.1	0.2 ± 0.0	0.2 ± 0.1	2.2 ± 0.5	0.2 ± 0.0	1.8 ± 0.8	1.2 ± 0.2
7.	Terpinene-4-ol	Ter-4ol	1.1 ± 0.3	0.4 ± 0.1	0.6 ± 0.0	3.0 ± 0.8	0.2 ± 0.1	0.3 ± 0.1	0.4 ± 0.1	0.2 ± 0.2
8.	Aromadendrene	aro	1.6 ± 0.8	0.1 ± 0.0	0.5 ± 0.2	0.4 ± 0.0	2.0 ± 0.8	0.2 ± 0.0	3.6 ± 1.2	2.1 ± 1.4
9.	tr-Pinocarveol	tr-pin	2.0 ± 0.8	1.0 ± 0.2	1.2 ± 0.1	0.8 ± 0.2	4.6 ± 0.6	1.0 ± 0.2	7.0 ± 2.5	4.3 ± 1.0
10.	Cryptone	cry	-	-	tr ± 0.1	20.9 ± 1.3	-	-	0.1 ± 0.1	-
11.	α-Terpineol	*α*-ter	2.2 ± 0.2	10.3 ± 1.1	5.4 ± 0.9	0.8 ± 0.2	0.6 ± 0.3	8.7 ± 2.5	1.3 ± 0.3	1.6 ± 0.7
12.	Verbenone	ver	-	-	tr ± 0.1	0.9 ± 0.2	-	-	0.1 ± 0.1	-
13.	Phellandral	phe	-	0.2 ± 0.2	-	6.6 ± 0.4	0.2 ± 0.2	-	-	-
14.	Cuminal	cum	-	0.1 ± 0.0	-	6.6 ± 0.6	-	-	-	-
15.	*tr*-p-Mentha-1.7. 8dien-2-ol	tr-p-men	0.5 ± 0.1	1.0 ± 0.2	0.6 ± 0.0	0.5 ± 0.1	1.0 ± 0.1	0.2 ± 0.1	0.4 ± 0.1	0.8 ± 0.1
16.	*p*-Cymen-8-ol	*p*-cym	0.1 ± 0.0	0.1 ± 0.0	0.1 ± 0.0	2.9 ± 0.6	0.1 ± 0.0	tr ± 0.1	0.1 ± 0.0	0.1 ± 0.1
17.	*cis*-p-Mentha-1. 7. 8dien-2-ol	*cis*-men	0.4 ± 0.2	1.0 ± 0.2	0.6 ± 0.0	0.1 ± 0.1	1.0 ± 0.1	0.3 ± 0.2	0.4 ± 0.1	0.8 ± 0.1
18.	Caryophyllene oxide	car-ox	0.1 ± 0.0	tr ± 0.1	0.1 ± 0.1	1.7 ± 0.2	tr ± 0.1	0.1 ± 0.1	0.1 ± 0.0	tr ± 0.1
19.	Epiglobulol	epi	0.4 ± 0.2	0.1 ± 0.1	0.1 ± 0.0	tr ± 0.1	1.0 ± 0.2	0.1 ± 0.0	1.2 ± 0.3	1.1 ± 0.6
20.	Globulol	glo	1.7 ± 1.4	0.6 ± 0.2	1.1 ± 0.3	0.8 ± 0.2	5.4 ± 1.2	0.6 ± 0.2	6.2 ± 0.9	5.8 ± 2.9
21.	Viridiflorol	vir	0.7 ± 0.3	0.2 ± 0.1	0.4 ± 0.1	1.0 ± 0.3	0.8 ± 0.2	0.2 ± 0.1	1.1 ± 0.1	0.8 ± 0.4
22.	Spahulenol	spa	0.1 ± 0.1	0.2 ± 0.1	0.5 ± 0.3	3.2 ± 0.9	0.1 ± 0.0	1.0 ± 0.1	0.9 ± 0.1	0.1 ± 0.1
23.	Carvacrol	car	0.4 ± 0.1	0.1 ± 0.0	0.1 ± 0.0	1.7 ± 0.3	0.1 ± 0.0	0.2 ± 0.0	0.2 ± 0.1	0.3 ± 0.3
24.	*α*-Eudesmol	*α*-eud	1.1 ± 0.6	-	0.4 ± 0.1	-	0.1 ± 0.0	0.3 ± 0.1	0.1 ± 0.0	0.2 ± 0.1
25.	*β*-Eudesmol	*β*-eud	3.0 ± 1.9	0.2 ± 0.0	0.6 ± 0.1	0.1 ± 0.2	tr ± 0.1	0.3 ± 0.1	0.1 ± 0.0	0.1 ± 0.2

^a^ Values are means ± SD of triplicate determination.

^b^*E.maid = E. maidenii, E. cin = E. cinerea, E. Sid = E. sideroxylon, E. odo = E. odorata, E. bic = E. bicostata, E. leh = E. lehmannii, E. ast = E. astringens, E. leuc = E. leucoxylon.*

### Principal components analysis (PCA) and hierarchical cluster analysis (HCA)

**Figure 1:**
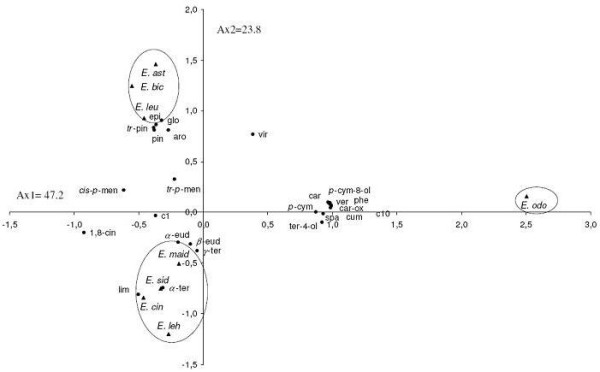
**ACP of the 26 major components of eight eucalyptus essential oils.** For the abbreviation of *Eucalyptus* species (▴) and components (●) see Table 1.

**Figure 2:**
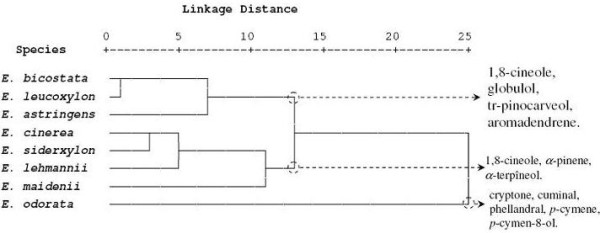
**Dendrogram obtained by hierarchical cluster analysis based on the Euclidean distance between groups of leaf essential oils of eight Tunisian *Eucalyptus* species.** Components that characterise the major sub groups, considered as chemotypes, are indicated.

The yield content of the 25 selected component was significantly different (*p* < 0.05) among species (Table 1). These 25 components were used for the PCA and the HCA analysis. The PCA horizontal axis explained 47.2% of the total variance while the vertical axis a further 23.80% (Figure 1). The HCA based on the Euclidean distance between groups indicated three specie groups (A, B and C) (with a dissimilarity of 11.0) (Figure 2), identified by their essential oil chemotypes. Group A clearly stood out forming a separate group in the PCA (Figure 1) and a deep dichotomy in the HCA (Figure 2). It was correlated positively with the axes 1 and 2. Groups A and B were negatively correlated, their separation was mainly due to axis 2. Group A species reduced to *E. odorata*, the essential oil of which was characterized by the highest mean percentage of cryptone (20.9 ± 1.3%), cuminal (6.6 ± 0.6%), phellandral (6.6 ± 0.4%), verbenone (0.9 ± 0.2%), *p*-cymen-8-ol (2.9 ± 0.6%), sapthulenol (3.2 ± 0.9%), carvacrol (1.7 ± 0.3%), *p*-cymene (16.7 ± 5.2%), terpinene-4-ol (3.0 ± 0.8%), caryophyllene oxyde (1.7 ± 0.2%), viridiflorol (4.5 ± 1.6%) and by the lowest level of 1,8-cineole (4.5 ± 1.6%). Group B, made up of *E. maidenii*, *E. lehmannii*, *E. sideroxylon* and *E. cinerea*, has essential oils characterized by the highest amount of limonene (3.1 ± 0.2 to 4.4 ± 0.2%), α-terpineol (2.2 ± 0.3% for *E. maidenii* to 10.3 ± 1.1% for *E. cinerea*). The PCA showed that the variation between these species is mainly due to the variation of 1,8-cineole content (57.8 ± 1.9% for *E. maidenii* to 70.4 ± 2.5 for *E. cinerea*) and of *α*-pinene (4.5 ± 0.7% for *E. cinerea* to 17.6 ± 7.5% for *E. lehmannii*). Group C, consisting of *E. astringens*, *E. leucoxylon* and *E. bicostata*, has essential oils distinguished by their highest mean percentage of epiglobulol (1.0 ± 0.2 - 1.2 ± 0.3%), globulol (5.4 ± 1.2 - 6.2 ± 0.9%), *trans*-pinocarveol (4.3 ± 1.0 to 7.0 ± 2.5%), aromadendrene (2.0 ± 0.8 - 3.6 ± 1.2%) and pinocarvone (1.2 ± 0.2 - 2.2 ± 0.5%). The *E. bicostata* and *E. leucoxylon* oils differed from *E. astringens* oil by their richness in 1,8-cineole (68.0 ± 5.3, 59.2 ± 10.1%, respectively) and their poverty in *α*-pinene (3.7 ± 1.2, 7.8 ± 2.3%, respectively) against 42.0 ± 5.9% and 22.0 ± 6.0%, respectively for the latter species.

As in the present study, *E. cinerea*, *E. sideroxylon*, *E. bicostata*, *E. maidenii*, *E. leucoxylon*, *E. lehmannii* and *E. astringens* have been reported to contain 1,8-cineole as a major component [26-30]. It was also reported that *E. cinerea* grown in Morocco contained a higher mean percentage of 1,8-cineole (87.8%) than that from Tunisia (70.4-2.5%) [27], whereas *E. sideroxylon* acclimated in Tunisia was richer in 1,8-cineole (69.2-0.6%) than that from Congo (59.9%) [31]. The Tunisian *E. astringens* essential oil had the same chemotype as that extracted from leaves picked from Moroccan tree plantations with an important difference in their mean percentages. Both of them were different from the *Australian E. astringens* essential oil [32], which was represented by *β*-caryophyllene (14.75%), *p*-cymene (17.72%), and *α*-pinene (12.53%). The same principal components were also observed in *E. leucoxylon* essential oil [33,34], whereas the essential oil obtained from Iran was significantly richer in 1,8-cineole (89.8%) than that from Tunisia [33].

### Antibacterial activity

**Figure 3:**
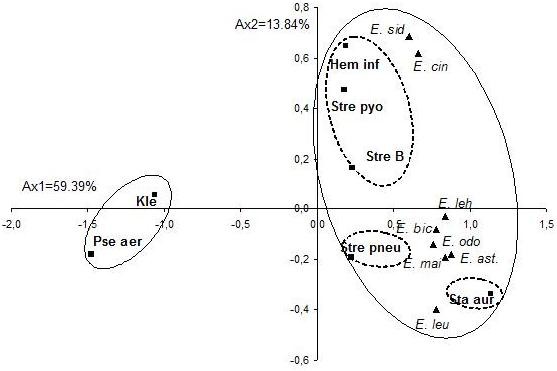
**PCA of the antibacterial activity of 0*8 Eucalyptus* essential oils species against seven clinical bacteria (■).** For the abbreviation of the *Eucalyptus* species (▴) see Table 1.**■**) *Hem inf = Haemophylis influenza, kle = Klebsiella pneumoniae = Pse aer: Pseudomans aeruginosa, sta aur = Staphylococcus aureus, Stre B = Streptococcus agalactiae, Stre pneu = Streptococcus pneumoniae, Strep pyo = Streptococcus pyogenes*.

**Figure 4:**
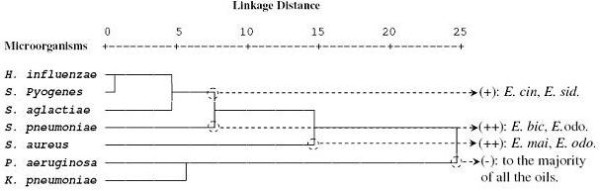
**Dendrogram obtained by hierarchical cluster analysis based on the Euclidean distance between groups of the antibacterial activities of leaf essential oils of eight Tunisian *Eucalyptus* species.** For the abbreviation of the *Eucalyptus* species (▴), see Table 1.

The essential oils were tested for their putative antibacterial activity against seven bacterial isolates represented by 81 strains (Table 2). As shown in Table 2, *E. odorata* oil possessed the best activity against *S. aureus* (27.4 ± 10.7 mm, zdi), followed by *S. agalactiae* (19.4 ± 5.6 mm, zdi), *H. influenzae* (19.2 ± 9.6 mm, zdi), *S. pyogenes* (19.0 ± 0.0 mm, zdi) and *S. pneumoniae* (17.4 ± 4.1 mm, zdi). *E. maidenii* oil showed a relatively good activity against *S. aureus* (22.8 ± 6.8 mm, zdi). To evaluate the correlation between the antibacterial activities and the essential oils of the eight *Eucalyptus* species, all the mean values of the zone diameters inhibition were subject to the PCA and the HCA analysis. The statistical analysis of the antibacterial activities of the oils showed a significant difference among *Eucalyptus* species oils and the tested bacterial strains (*p* < 0.05). The PCA horizontal axis explained 59.39% of the total variance, while the vertical axis a further 13.84% (Figure 3). The HCA showed two species groups (I and II), identified by their bacteria growth inhibition with a dissimilarity ≥ 15.0 (Figure 4). When the dissimilarity was ≥ 5.0, group II was divided into three subgroups (II_a_, II_b_ and II_c_). The horizontal axis permitted the separation of group I from group II, however axis II separated all the species of the group II into three subgroups. Group I, limited to the Gram negative (Gram(−)) bacteria, *P. aeruginosa* and *K. pneumoniae*, forms a deep dichotomy within the HCA analysis and a clearly separated group in the PCA analysis. These two strains were the most resistant to the majority of *Eucalyptus* essential oils with zdi < 7.1 mm for *P. aeruginosa* and 10.7 ± 1.5 mm for *K. pneumoniae*. Compared to the growth inhibition zone produced by ciprofloxacin against *P. aeruginosa* (34.7 ± 5.0 mm, zdi) and *K. pneumoniae* (32.4 ± 2.9 mm, zdi). Subgroup Ia was limited to *S. aureus* which was characterized by the highest sensitivity to *E. maidenii* and *E. odorata* oils (22.8 ± 6.8 and 27.4 ± 10.7 mm, zdi, respectively). This high sensitivity could be due to the disposition of *E. maidenii* and *E. odorata* oils with a relatively high mean percentage of the monoterpene hydrocarbons *p*-cymene (7.4 ± 2.9, 16.7 ± 5.2%, respectively). Previous studies have reported the high sensitive character of *S. aureus* to essential oils with a high content of *p*-cymene [35]. In addition, other researchers reported that this sensitivity of *S. aureus* was due to the single layer wall of the bacteria [36]. Comparing these results with those obtained with antibiotics, *E. odorata* essential oil produced a similar inhibition to that produced by gentamicin, erythromycin, vancomycin and benzylpenicillin (29.6 ± 6.2, 29.9 ± 5.1, 25.3 ± 4.4 and 24.5 ± 7.5 mm, dzi, respectively). However, this activity remained lower than that produced by fosfomycin (34.3 ± 11.1 mm, dzi). Sub group IIb represented by *S. pneumoniae*, showed a particular sensitivity to *E. odorata* and *E. bicostata* essential oils (17.4 ± 4.1 and 17.0 ± 4.0 mm, zdi). This inhibition remained lower than that produced by its specific antibiotics with zone diameters inhibition ranging from 26.3 ± 12.0 mm (erythromycin) to 35.6 ± 5.5 mm (fosfomycin). *E. lehmannii*, *E. sideroxylon* and *E. cinerea* oils did not show significant antibacterial activities with inhibition zones of 9.8 ± 2.4, 10.7 ± 2.5 and 11.5 ± 2.8 mm, respectively. Subgroup IIc, consists of *Streptococcus B*, *S. pyogenes* and *H. influenzae*. These strains were separated from all the others and correlated positively with the two axes and with *E. cinerea* and *E. sideroxylon*, the essential oils of which were characterized by a comparable activity against the previous bacterial strains, with inhibition zone diameters varying from 11.6 ± 1.4 mm to 13.0 ± 6.3 mm. Their activities were considered relatively as being lower than the tested antibiotics such as rifamicine and ampicilline. However *E. odorata* oil, which was removed from this group, showed the best activity against these bacterial strains with inhibition zone diameters varying from 17.4 ± 4.1 mm for *S. pneumoniae* to 19.4 ± 5.6 for *Streptococcus B,* but it remained much lower than that produced by their specific antibiotics*.* The MIC was performed for oils which have produced an inhibition ≥ 17 mm for clinical bacterial strains such as *H. influenzae* (reference 160), *S. agalactiae* (reference 3) *S. pyogenes* (reference 545) and *S. aureus* (reference 278). The result of their MIC was listed in Table 3. *E. odorata and E. bicostata* oils were characterized by the lowest MIC for *Hemophylis influenzae* (reference 160) (0.306 mg/mL), followed by *S. agalactiae* (reference 3) (10.4 mg/mL). These results were confirmed by the disc diffusion method. The highest MIC against *S. aureus (*reference 278) was shown for the oils of *E. bicostata* (169 mg/mL), *E. odorata* (156.6 mg/mL) and *E. maidenii* (151.8 mg/mL). This finding was in contradiction to results obtained by the disc diffusion method. According to the classification of Schaechter et al. (1999) [37] and Soro et al. (2010) [38], *E. odorata, E. bicosta* and *E. maidenii oils* were considered bactericidal (MBC/MIC < 4) against the tested strains, however the two first oils showed a better bactericidal activity against *H. influenzae* (reference 160) and *S. aureus* (reference 278) than that obtained with *S. pyogens* (reference 545) and *S. aglatctiae* (reference 3). 

**Table 2:** Diameter of the inhibition of respiratory bacterial growth by essential oils and by antibiotics*^)^

**Essential oils**	**Tested microorganisms (number of strains)**						
	***H. influenzae*(11)**	***K. pneumoniae*(13)**	***P. aeruginosa*(10)**	***S. aureus*(17)**	***S. agalactiae*(9)**	***S. pneumoniae*(19)**	***S. pyogenes*(4)**
*E. maidenii*	14.5 ± 5.7^bc·)^	10.7 ± 1.5^ab^	7.1 ± 1.5^a^	22.8 ± 6.8^d^	13.8 ± 2.2^bc^	13.0 ± 2.6^bc^	15.5 ± 4.0^c^
*E. odorata*	19.2 ± 9.6^b^	10.8 ± 1.2^a^	6.0 ± 0.0^a^	27.4 ± 10.7^c^	19.4 ± 5.6^b^	17.4 ± 4.1^b^	19.0 ± 0.0^b^
*E. lehamnnii*	11.5 ± 3.3^bc^	6.8 ± 0.7^a^	6.5 ± 0.8^a^	14.2 ± 4.8^c^	11.1 ± 1.4^b^	9.8 ± 2.4^b^	11.5 ± 1.7^bc^
*E. leucoxylon*	8.1 ± 2.2^a^	6.6 ± 1.1^a^	6.0 ± 0.0^a^	16.4 ± 5.4^c^	12.9 ± 1.8^b^	14.4 ± 3.0^bc^	9.0 ± 0.0^a^
*E. bicostata*	13.6 ± 5.0^b^	7.1 ± 1.4^a^	6.0 ± 0.0^a^	15.6 ± 2.8^bc^	13.8 ± 2.8^b^	17.0 ± 4.0^c^	13.0 ± 0.0^b^
*E. astringens*	13.0 ± 3.8^bc^	7.1 ± 1.6^a^	6.0 ± 0.0^a^	15.5 ± 5.2^c^	12.0 ± 3.2^b^	12.3 ± 12.3^bc^	12.5 ± 1.7^bc^
*E. cinerea*	13.0 ± 6.3^b^	8.5 ± 2.2^a^	6.2 ± 0.6^a^	12.2 ± 2.8^b^	11.6 ± 1.4^b^	11.5 ± 2.8^b^	12.5 ± 1.7^b^
*E. sideroxylon*	12.3 ± 8.4^b^	8.1 ± 1.8^a^	7.0 ± 1.5^a^	13.7 ± 3.8^b^	12.3 ± 1.6^b^	10.7 ± 2.5^ab^	12.0 ± 0.0^b^
**Reference antibiotics**							
Ampicillin	30.5 ± 6.1^c^	NT	NT	NT	NT	NT	NT
Benzylpenicillin (Penicillin G)	NT	NT	NT	24.5 ± 7.5^a^	NT	NT	NT
Ceftazidime	NT	22.3 ± 7.5^a^	23.8 ± 5.6^a^	NT	NT	NT	NT
Cehotaxime	20.7 ± 6.0^b^	NT	NT	NT	NT	NT	NT
Ciprofloxacin	NT	32.4 ± 2.9^b^	34.7 ± 5.0^b^	NT	NT	NT	Nt
Erythromycin	19.9 ± 4.2^b^	NT	NT	29.9 ± 5.1^ab^	29.2 ± 4.2^b^	26.3 ± 12.0^a^	29.0 ± 0.0^c^
Fosfomycin	NT	22.1 ± 2.9^a^	NT	34.3 ± 11.1^c^	27.9 ± 5.9^ab^	35.6 ± 5.5^c^	24.5 ± 0.8^a^
Gentamicin	NT	22.9 ± 5.0^a^	NT	29.6 ± 6.2^ab^	NT	NT	NT
Imipenem	NT	NT	24.6 ± 6.6^a^	NT	NT	NT	NT
Levofloxacin	NT	NT	NT	NT	24.8 ± 2.8^a^	27.3 ± 3.5^ab^	25.0 ± 1.5^ab^
Lincomycin	11.3 ± 1.8^a^	NT	NT	NT	NT	NT	NT
Oxacillin	NT	NT	NT	NT	33.6 ± 4.2^c^	31.7 ± 11.9^abc^	30.0 ± 0.0^c^
Piperacillin	NT	NT	25.3 ± 7.3^a^	NT	NT	NT	NT
Rifamycin	23.9 ± 4.7^b^	NT	NT	NT	34.4 ± 4.3^c^	37.5 ± 8.1^c^	31.5 ± 0.8^d^
Tetracyclin	29.2 ± 2.6^c^	NT	24.4 ± 6.1^a^	NT	NT	NT	NT
Vancomycin	NT	NT	Nt	25.3 ± 4.4^a^	26.3 ± 3.4^ab^	33.0 ± 3.5^bc^	26.0 ± 2.0 ^b^

^*^Values are means (mm. ±SD) of triplicate determination.

·) Values with different letters differ significantly by Duncan's multiple range test (*p* < 0.05).

NT = Not tested.

**Table 3:** Minimal Inhibition concentration (MIC), minimal bactericidal concentration (MBC) and ratio MBC/MIC of the most effective oils against respiratory microorganisms

**Essential oils**	**Tested microorganisms**				
		***H.inf*160**^**a)**^	***Stre B3***	***S.pyo*545**	***S.aur*278**
*E. odorata*	MIC	0.306^b)^	10.417	9.792	156.667
	MBC	0.306	20.834	19.584	156.667
	MBC/MIC	1	2	2	1
*E. bicostata*	MIC	0.331	10.562	NT	169.000
	MBC	0.331	21.125	NT	169.000
	MBC/MIC	1	2	NT	1
*E. maidenii*	MIC	NT	NT	NT	151.867
	MBC	NT	NT	NT	151.867
	MBC/MIC	NT	NT	NT	1

^a)^: *H. inf 160* = *Hemophylus influenza (160); Strep B3 = Streptococus B3* = *Streptococcus agalactiae*3; *S.pyo*545 = *Streptoccus pyogenes; S.aur*278 = *Staphylococcus aureus*.

^b)^: values were expressed as mg/ml; NT = Not tested.

### Antifungal activity

Seven *Eucalyptus* oils were tested for their antifungal and anti-yeast activities. The result of their average percentages inhibition was listed in Table 4. Their variance analysis showed a highly significant effect for all oils (*p* < 0.05), except for *E. odorata* and *E. cinerea* oils (*p* > 0.05).

**Table 4:** Antifungal activity of seven* Eucalyptus* essential oils*^)^

**Essential oils**	**Tested microrganisms**				
	***Trichophyton rubrum***	***Trichophyton soudanense***	***Microsporum canis***	***Scopulariopsis brevicaulis***	***Candida albicans***
*E. maidenii*	31.9 ± 2.1^a·)^	66.8 ± 17.1^b^	53.9 ± 0.0^b^	22.8 ± 2.1^a^	60.0 ± 0.0^b^
*E. odorata*	100.0 ± 0.0^b^	100.0 ± 0.0^b^	100.0 ± 0.0^e^	69.7 ± 0.0^a^	100.0 ± 0.0^b^
*E. sideroxylon*	30.4 ± 0.0^bc^	29.0 ± 6.4^b^	68.1 ± 5.1^d^	12.2 ± 4.3^a^	40.0 ± 0.0^c^
*E. bicostata*	28.9 ± 2.1^a^	23.0 ± 6.4^a^	59.2 ± 2.5^b^	28.9 ± 2.1^a^	80.0 ± 0.0^c^
*E. astringence*	37.9 ± 2.1^b^	38.1 ± 2.1^b^	61.0 ± 0.0^c^	22.8 ± 10.7^a^	60.0 ± 0.0^c^
*E. lehmannii*	27.3 ± 0.0^b^	39.6 ± 4.3^c^	34.3 ± 2.5^c^	0.1 ± 0.0^a^	40.0 ± 0.0^c^
*E. cinerea*	24.3 ± 0.0^ab^	41.1 ± 15.0^cd^	55.6 ± 12.6^d^	4.6 ± 6.4^a^	40.0 ± 0.0^cd^
Pevaryl (std)	100.0 ± 0.0^a^	100.0 ± 0.0^a^	100.0 ± 0.0^a^	100.0 ± 0.0^a^	100.0 ± 0.0^a^

^*)^ Values were expressed as a percentage of inhibition ± SD at the concentration 1000 ppm of triplicate determination.

·) Values with different letters differ significantly by Duncan's multiple range test (*p* < 0.05) std: standard for antifungal activity. *E.*: *Eucalyptus*.

**Figure 5:**
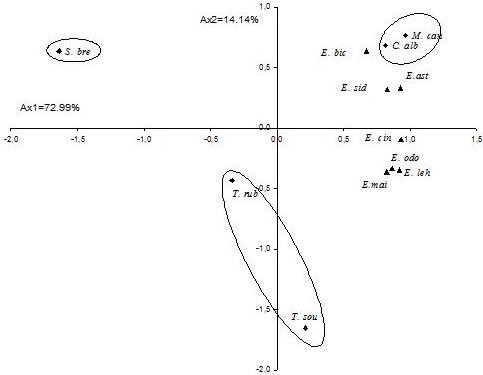
**PCA of the antifungal activity of 0 *8 Eucalyptus*essential oils species against five fungi (**♦**):* T. rub *** **=** *** Trichophyton rubrum, T. sou = Trichophyton soudanense, M. can = Microsporum canis; S. bre = Scopulariopsis brevicaulis, C. alb = Candida albicans*.** For the abbreviation of the *Eucalyptus* species (▴) (see Table 1).

**Figure 6:**
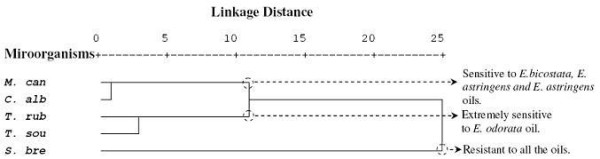
**Dendrogram obtained by hierarchical cluster analysis based on the Euclidean distance between groups of the antifungal activities of leaf essential oils of seven Tunisian *Eucalyptus* species.** For the abbreviation of the fungi strains, see Figure 5.

The PCA horizontal axis explained 73% of the total variance, while the vertical axis a further 14.15% (Figure 5). The HCA indicated three groups of species (A, B and C) with a dissimilarity ≥ 5.0 (Figure 6).

Group A, limited to *Scopulariopsis brevicaulis*, correlated negatively with axis 1 and with the group C. This species was distinguishable in the PCA and formed a distinct group. It was considered as being the most resistant to all the tested oils. Group B was formed by *Trichophyton soudanense* (correlating positively and negatively with axis 1 and 2, respectively) and by *Trichophyton rubrum* (correlating negatively with the two axes). These strains were distinguished by their highest sensitivity to *E. odorata* oil (100% of inhibition); however, the variability between these two fungi strains was mainly due to the higher sensitivity of *Trichophyton soudanense* to *E. lehmannii* and *E. maidenii* oils than that of *Trichophyton rubrum* and also to the resistance of *Trichophyton soudanense* to *E. cinerea* oil. Group C, which was formed by the yeast *Candida albicans* and by the fungi *Microsporum canis*, correlated positively with the two axes and was characterized by a relatively high sensitivity to *E. bicostata*, *E. astringens* and *E. sideroxylon*; however, these strains showed a higher resistance to the other oils.

According to the chemical composition of these essential oils, the antifungal activity was not related to the high content of one chemical compound, rather than to synergic effects between major and minor components. For example, 1,8-cineole, which was discussed above as the principal component of the most essential oils, did not correlate with the high antifungal activity because *E. c*i*nerea* (70.4 ± 2.5%)*, E. leucoxylon* (59.2 ± 10.1%)*, E. lehmanii* (56.6 ± 4.3%)*,* and *E. maidenii* (57.8 ± 1.9%) showed a lower antifungal activity than *E. bicostata*, *E. astringens* and *E. sideroxylon.* Additionally, the essential oils of *E. bicostata*, *E. astringens,* characterized by the highest content of pinocarvone (2.2 ± 0.5 and 1.8 ± 0.8%, respectively), tr-pinocarveol (4.6 ± 0.6 and 7.0 ± 2.5%, respectively) and globulol (5.4 ± 1.2 and 6.2 ± 0.9%, respectively), showed a lesser antifungal activity than the essential oil of *E. sideroxylon*.

### Cytotoxicity assay

The cytotoxicity effect of the eight *Eucalyptus* essential oils on Vero cell lines varied significantly within species (Table 5). Vero cells were resistant to the essential oils of *E. maidenii*, *E. sideroxylon* and *E. cinerea* with CC_50_ values of 253.5, 247.3 and 204.5 mg/mL, respectively. The essential oils of *E. odorata*, *E. leucoxylon*, *E. lehmannii*, *E. astringens* and *E. bicostata* demonstrated a different behavior and their cytotoxicity increased considerably with CC_50_ varying from 6.2 to 16 mg/mL. We did not notice any clear correlation between the chemical composition of the tested oils with the results of the cytotoxic effect and further investigation needs to be undertaken. However, the lowest cytotoxicity was observed with oils having a high content of 1,8-cineole but with a moderate amount of *α*-pinene and limonene such as those of *E. maidenii*, *E. sideroxylon* and *E. cinerea*. The high cytotoxic effect was shown with *E. lehmannii*, *E. astringens* oils, which were characterized by a higher mean percentage of the monoterpene *α*-pinene and by a moderate mean percentage of 1,8-cineole. The present result was confirmed by Setzer et al. (2006) [39], who demonstrated that the monoterpene hydrocarbons *α*-pinene had a stronger cytotoxicity activity against Hs 578 T and Hep-G2 cell lines than 1,8-cineole. It was also observed that a synergic effect among the oxygenated sesquiterpens globulol and viridiflorol and the monterpene hydrocarbons *α*-pinene of *E. astringens*, *E. lehmannii* and *E. leucoxylon* oils could make the cell lines more sensitive. On the other hand, the significant high cytotoxicity of *E. odorata* oil could be explained by the latter’s lack of 1,8-cineole and its richness in the ketone cryptone, the monoterpene hydrocarbons *p*-cymene and in theses aldehydes: phellandral and cuminal [40]. This cytotoxicity effect could be due to the synergetic effect of the previous main constituents of this essential oil. Compared to the previous studies, the cytotoxicity of our studied essential oils was very low CC_50_ > 20 μg/mL [21]. Therefore they could be considered as being safe for use at non cytotoxic concentrations. 

**Table 5:** **Cytotoxicity effect and antiviral activity of eight* Eucalyptus* essential oils**

**Essential oils**	**СС**_50_^*)^** (mg/mL, ± SD)**	**Concentrations (mg/mL)**	**Antiviral activity**					
			**Pre-treatment of cells**	**IC**_**50**_** (mg/ml)**	**SI**	**Pre-treatment of virus**	**IC**_**50**_** (mg/ml)**	**SI**
*E. cin*	204.5 ± 0.35^b•)^	150	66.5	131^b^	1.56^a^	71	102^c^	2^a^
		75	0			54		
		37.5	0			0		
*E. sid*	247.3 ± 0.9^c^	150	0	Nd	Nd	0	Nd	Nd
		75	0			0		
		37.5	0			0		
*E. mai*	253.5 ± 0.35^c^	150	29	233.5^c^	1.08^a^	62.5	136.5^d^	1.85^a^
		75	0			0		
		37.5	0			0		
*E. leh*	15 ± 00.0^a^	15	0	Nd	Nd	0	Nd	Nd
		7.5	0			0		
		3.75	0			0		
*E. bic*	16 ± 00.0^a^	15	0	4.8^a^	3.33^b^	0	0.7^a^	22.8^b^
		7.5	66.5			87.5		
		3.75	37.5			83		
*E. ast*	15.7 ± 0.14^a^	15	0	Nd	Nd	83	8.4^b^	1.86^a^
		7.5	0			46		
		3.75	0			25		
*E. leu*	14.9 ± 0.07^a^	15	0	Nd	Nd	0	Nd	Nd
		7.5	0			0		
		3.75	0			0		
*E. odo*	6.2 ± 0.14^a^	6	0	Nd	Nd	0	Nd	Nd
		3	0			0		
		1.5	0			0		

^*)^ Values were expressed as a cytotoxic concentration ± SD of duplicate determination.

·) Values with different letters differ significantly by Duncan's multiple range test (*p* < 0.05).

Nd: Not determined.

### Antiviral activity

In order to elucidate the mode of antiviral action and to identify the target site, cells were pre-treated with essential oils before viral infection (pre-treatment of cells) and the virus was incubated with essential oils before cell inoculation (pre-treatment of virus). All samples tested were used at their maximum non-cytotoxic concentrations (Table 5). The essential oils of *E. sideroxylon*, *E. lehmannii*, *E. leucoxylon* and *E. odorata* showed no inhibition of viral infection, whereas the most significant antiviral activity was shown with the essential oils of *E. bicostata* (IC_50_ = 0.7 - 4.8 mg/mL) and *E. astringens* (IC_50_ = 8.4 mg/mL), followed by essential oils of *E. cinerea* (IC_50_ = 102–131 mg/ml) and *E. maidennii* (IC_50_ = 136.5 - 233.5 mg/mL). The selectivity index describes the ratio between the cytotoxic and the antiviral activity of a tested sample. The virus pretreatment with *E. bicostata* essential oil showed a better antiviral activity (IC_50_ = 0.7 mg/mL, SI = 22.8) than cell-pretreatment (IC_50_ = 4.8 mg/mL, SI = 3.33). The essential oil of *E. astringens* showed an antiviral activity only when incubated with a virus prior to cell infection. This activity was dose-dependent and the antiviral activity decreased with the diminishing essential oil concentration. *E. cinerea* and *E. maidenii* essential oils showed an antiviral activity at a concentration of 150 mg/mL when incubated with cells. This activity increased significantly at the same concentration when the sample was incubated with a virus prior to infection.

According to these results, no correlation was found between the chemical composition and the antiviral assay. Therefore the activity of the tested essential oils could be due to a synergism between the major and the minor components. Altogether, the essential oils used in the present study exhibited the best antiviral effect when incubated with a virus. From the presented results, *E. odorata* oil was associated with *E. maidenii*, *E. bic*ostata, *E. lehamnnii*, *E. astringens* and *E. leucoxylon* oils in both the antibacterial PCA and HCA. However, it remained associated with *E. maidenii* and *E. lehmannii* in the antifungal PCA analysis. Therefore, the biological activity has allowed the association of different chemotypes in the same group producing a similar biological activity. This allowed us to deduce that the global biological activity of these oils was mainly due to an addition or a synergism effect between the major and the minor components. This was confirmed by Paster et al. (1995) [41]. The best activity was recorded with *E. odorata* oil against the majority of the microbial strains with inhibition zone diameters almost equal to those produced by erythromycin against clinical strains *H. inflenzae*, *S. aureus*, and to the Pevaryl against the three dermatophytes fungi and the *Candida albicans*. This property could be explained by the richness of *E. odorata* oil in *p*-cymene (16.7 ± 5.2%), terpinene-4-ol (3.0 ± 0.8%), spathulenol (3.2 ± 0.9%), carvacrol (1.7 ± 0.3%), caryophyllene oxide (1.7 ± 0.2%), *p*-cymene-8-ol (2.9 ± 0.6%), verbenone (0.9 ± 0.2%), viridiflorol (1.0 ± 0.3%), phellandral (6.6 ± 0.4%), cuminal (6.6 ± 0.6%) and cryptone (20.9 ± 1.3%), and by its poverty in 1,8-cineole (4.5 ± 1.6%). It appeared that all the strains were resistant to oils rich in the latter component which varied from 42.0 ± 5.9% for *E. astringens* to 70.4 ± 2.5% for *E. cinerea*. On the other hand, oils characterized by a small quantity of 1,8-cineole and by a medium mean percentage of the monoterpene, *p*-cymene, the ketone, cryptone, the aldehydes, phellandral and cuminal were more active. These results were confirmed by Mulyaningsih et al. (2010) [42], who demonstrated that the antimicrobial activity of pure 1,8-cineole was inferior to the totality of *E. globulus* fruit oil, considered poor in the latter component and rich in aromadendrene and globulol. Dorman and Deans (2000), also confirmed that the antimicrobial activity increased with oils rich in aldehydes, ketones and phenols [43]. *E. maidenii* oil was characterized by a relatively high mean percentage of 1,8-cineole (57.8 ± 1.9%), *p*-cymene (7.4 ± 2.9%), *α*-pinene (7.3 ± 0.7%), limonene (3.1 ± 0.2%), *α*-terpineol (2.2 ± 0.2%), the activity of which occupied the second position after *E. odorata* oil against all the bacterial strains (7.1 ± 1.5 mm, zdi, for *P. aeruginosa* to 22.8 ± 6.8 mm, zdi, for *S. aureus*) and also against the fungal strains with an inhibition percentage varying from 22.8 ± 2.1% for *Scopulariopsis brevicaulis* to 66.8 ± 17.1% for *Trichophyton soudanense*. However, oils of *E. sideroxylon* and *E. cinerea,* which were distinguished by the highest levels in 1,8-cineole, were less active against the tested microorganisms than the majority of the remaining oils. The present finding was in contradiction with previous studies reporting that 1,8-cineole had strong antimicrobial properties against many important pathogens [44,45]. It seems that the activity of this chemical compound was inhibited by other minor components. Further investigations need to be carried out to better understand the present issue.

According to the study of Claudio et al. (2008), the essential oil of *E. globulus* has shown a higher antibacterial activity against *Haemophilus influenzae* (28 mm, zdi) than the tested essential oils in the present study, whereas it possessed comparative inhibition activities against *S. pneumoniae* (15 mm vs 9.8-17.4 mm, zdi). However, our essential oils exhibited a better activity against *K. pneumoniae* (6.6-10.7 mm vs 0 mm, zdi), *S. aureus* (12.2-27.4 mm vs 2 mm, zdi), *S. agalactiae* (11.1-19.4 mm vs 3 mm, zdi) and *S. pyogenes* (9–19 mm vs 5 mm, zdi) [46]. Martin et al. (2010) have reported similar findings concerning the antibacterial activity of the essential oils of *E. dives* and *E. staigeriana* against *Pseudomonas aeruginosa* (7.7-9.1 mm vs 6–7.1 mm, zdi) [47].

We also noticed that *Microsporum canis* is more sensitive to *E. sideroxylon* oil than that of *E. cinerea*. This sensitivity could be attributed to the presence of a higher content of *α*-pinene and limonene and a lower percentage of *α*-terpineol in *E. sideroxylon* oil. The comparative study of the antibacterial activity result of the tested oils obtained by the disc diffusion method with the results obtained by micro-well dilution showed for some species a concordance of the two methods and discordance for others, especially against the Gram positive (Gram (+)), S*. aureus* (278). Compared to the activities produced by the tested antibiotics, all the essential oils were less active. An association of the most active oils together may moderate the activity against the most resistant strains.

## Conclusions

In conclusion, our study showed that the chemical PCA and HCA analysis separated all the species oils into three groups, each group constituted a chemotype; however, in the PCA and HCA analysis of the antibacterial activity, five groups and sub groups of bacterial strains were identified and separated by their sensitivity levels to the tested essential oils. *E. cinerea* of the group B was the richest species in 1,8-cineol. However, *E. odorata* oil of the group A was the richest in cryptone. The *Eucalyptus* essential oils activity varied significantly within species and within strains. In general, the strong antimicrobial activity was not related only to a high content of one major component such as 1,8-cineole, but also to the presence of moderate and minor compounds*.* The Gram (−) bacteria *P. aeruginosa* and *K. pneumoniae* were the most resistant to all the oils but more sensitive to the antibiotics, ciprofloxacin and ampicillin. Compared to antibiotics and antifungal fosfomycin, ampicillin, rifamycin and pevaryl, the clinical strains *S. aureus*, *H. influenzae*, *S. agalactiae*, *S. pyogenes*, *S.pneumoniae* and all the tested fungal strains were more sensitive to *E. odorata* oil. Taking into account all these results, *E. odorata* oil provide a promising product in the therapeutic application for the treatment of some respiratory bacterial infections and fungal diseases*.* The cytotoxicity of the tested oils on Vero cell lines varied significantly within species. *E. odorata* oil which was the richest species in the ketone cryptone, had the highest cytoxicity, whereas *E. maidenii, E. sideroxylon* and *E. cinerea* oils, which were characterized by the highest mean percentage of 1,8-cineole and by a moderate amount of α-pinene, were less toxic. *E. bicostata* oil had the best antiviral activity against coxsakievirus B3 whenever incubated with the Vero cell lines or with the virus. This essential oil may serve as a potential candidate against enterovirus infections. Further analyses need to be undertaken to test this essential oil on other viruses belonging to the genus enterovirus. However, *E. astringens* oil exhibited a significant antiviral activity only when incubated with the virus. This shows that this essential oil had a direct effect on the coxsakievirus B3. We could not elucidate the mode of action of these essential oils and their interactions with both cells and the virus remains to be elucidated.

## Abbreviations

E: Eucalyptus; GC: Gaz Chromatography; GC/MS: Gaz Chromatography coupled to the Mass Spectroscopy; HP: Hewlett-Packard; FID: Flame Ionization Detector; MH: Mueller–Hinton; MIC: Minimal Inhibition Concentration; MBC: Minimum Bactericidal Concentration; MTT: 3-[4,5-dimethylthiazol-2-yl]-2,5-diphenyl tetrazolium bromide; DMSO: Dimethyl Sulfoxide; OD: Optical density; CC50: The 50% cytotoxic concentration; IC50: The 50% inhibition concentration; PCA: Principal Components Analysis; HCA: Hierarchical Clusters Analysis; SI: Selectivity index; RI: Retention index; Gram(-): Gram negative; Gram(+): Gram positive.

## Competing interests

The authors declare that they have no competing interests.

## Authors’ contributions

AE and ZR carried out the studies, acquired the data, performed the data analysis, and drafted the manuscript. NA performed the cytotoxic and the antiviral assays. YBS played a major role in the experimental procedures of the antibacterial activities. SM and KB MA helped in the experimental procedure of the antifungal activity. FF and R.C offered the material such as (GC apparatus and the Pharmacopoeia European hydrodistilator apparatus) and revised the manuscript. FHS carried out the statistical analysis, and involved in the interpretation of the results and in the final revision of the work. MLK offered us the vegetable material.All authors read and approved the final manuscript.

## Pre-publication history

The pre-publication history for this paper can be accessed here:

http://www.biomedcentral.com/1472-6882/12/81/prepub
